# The sense of belonging reduces ingroup favoritism in children

**DOI:** 10.3389/fpsyg.2022.1059415

**Published:** 2022-12-01

**Authors:** Joo Hyang Park, Kyong-sun Jin

**Affiliations:** Department of Psychology, Sungshin Women’s University, Seoul, Republic of Korea

**Keywords:** children, belonging, ingroup favoritism, intergroup intervention, minimal group, prosocial behavior, sharing

## Abstract

Belonging is an important motive for intergroup behavior. Adults display pronounced ingroup favoritism when the sense of inclusion by an ingroup is decreased or threatened. The present study investigated whether ingroup belonging reduces ingroup favoritism in 6-year-old children in terms of costly sharing. Children were allocated to a novel group in a minimal-group paradigm. In two conditions, children played a brief ball-tossing game and were either included (ingroup-inclusion condition) or excluded (ingroup-exclusion condition) by their ingroup members. Children in a no-interaction condition did not have any interactions with the members of the ingroup. After this manipulation, we tested the extent to which children shared resources with ingroup and outgroup members. We found that children in the ingroup-exclusion and no-interaction conditions shared more resources with their ingroup member than their outgroup member, while children in the ingroup-inclusion condition shared equally with the ingroup and outgroup members. These results could inform interventions aimed at fostering positive intergroup relations.

## Introduction

Ingroup favoritism refers to the tendency to favor ingroups over outgroups in evaluations and actions. Individuals tend to evaluate ingroup members more positively than outgroup members, prefer ingroup members, allocate more resources to ingroup members, and selectively help ingroup members (e.g., Tajfel et al., 1971; Brewer, 1979; Killen and Turiel, 1998; Turiel, 1998; Hewstone et al., 2002; Tajfel and Turner, 2004; Levine et al., 2005; Fehr et al., 2008; Balliet et al., 2014). Ingroup favoritism is deeply rooted in evolutionary history, as group living has been critical to human survival. Our dependence on groups has at least two psychological consequences: we have a pervasive and fundamental motivation to belong to a group (Brewer, 1991; Baumeister and Leary, 1995; Fiske, 2010; Over, 2016) and we act in ways that support the group by caring for ingroup members and showing them loyalty (Tajfel et al., 1971; Shweder, 1997; Brewer, 1999; Tooby et al., 2006; Rai and Fiske, 2011; Graham et al., 2013; Baillargeon et al., 2015). Thus, one might expect the sense of belonging to a group to be associated with the tendency to be selectively prosocial with respect to the group (e.g., Brewer, 1991; Leary et al., 1995; Leary, 2005; Leonardelli et al., 2010). In the present study, we investigated the way in which the sense of belonging impacted ingroup favoritism in children. We proposed that children would show less ingroup favoritism when they had a strong sense of belonging to the ingroup.

The relationship between group belonging and ingroup favoritism has been demonstrated in adults. This line of research has mainly focused on the reactions of adults whose sense of inclusion by a group is lowered or threatened (e.g., people who are on the margins of a group or are excluded by the group). Adults in such situations often respond with relatively pronounced ingroup favoritism (Noel et al., 1995; Jetten et al., 2002; Williams et al., 2003; Vignoles and Moncaster, 2007), ingroup loyalty (Gómez et al., 2011), nonconscious mimicry of ingroup members (Lakin et al., 2008), ethnocentrism (Greitemeyer, 2012), and fundamentalist beliefs that are endorsed by the ingroup (Schaafsma and Williams, 2012). For example, in one study, peripheral group members (who presumably felt a need to assimilate) showed greater distinction in their attitudes toward their ingroup versus outgroup members than core group members (Noel et al., 1995), suggesting that threats to group inclusion motivate ingroup favoritism as a means of re-establishing belonging. These findings are consistent with the idea that individuals who are motivated to enhance their inclusionary status with respect to the group will attempt to display their value and worth as a group member, and that this may be achieved by favoring the ingroup over the outgroup (Leary et al., 1995; Leary, 2005).

The premise that threat to group belonging is an important motive for ingroup favoritism (Noel et al., 1995; Jetten et al., 2002) could inform interventions aiming to reduce intergroup bias in children. Recent findings indicate that ingroup favoritism emerges early in young children (Kinzler et al., 2007; Fehr et al., 2008; Olson and Spelke, 2008; Gummerum et al., 2009; Moore, 2009; Dunham et al., 2011; Paulus and Moore, 2014; Renno and Shutts, 2015; Yu et al., 2016; Sparks et al., 2017; Yazdi et al., 2020) and even in infants (e.g., Kinzler et al., 2007; Powell and Spelke, 2013; Jin and Baillargeon, 2017; Bian et al., 2018; Ting et al., 2019; Pun et al., 2021). These findings led developmental researchers to orient their efforts in a new direction and to develop targeted interventions to prevent the negative consequences of ingroup favoritism, including discrimination and prejudice. Strategies for reducing intergroup bias in children focus on moderating intergroup cognitions and emotions, for example, promoting interethnic friendships (Aboud et al., 2012), increasing empathy toward outgroup members (Sierksma et al., 2014, 2015; Abrams et al., 2015), empathizing with outgroup members (McLoughlin and Over, 2019), reading stories describing positive intergroup interactions (Cameron et al., 2006; Cameron and Rutland, 2006), and imagining interpersonal contact with outgroup members (Vezzali et al., 2012, 2015). However, harnessing this knowledge to achieve sustained positive outgroup cognitions and emotions can be challenging (Dixon et al., 2007; Dovidio et al., 2009; Vorauer and Sasaki, 2009; Brown et al., 2016; Walton and Yeager, 2020).

The present study investigated whether children’s sense of belonging to a group reduced ingroup favoritism. If threats to group inclusion motivate the expression of ingroup favoritism as a means of establishing belonging (Leonardelli et al., 2010), children who are completely accepted by their ingroup might be less likely to treat its members preferentially compared to children who experience rejection. To the best of our knowledge, only a few published experimental reports have investigated how group belonging influences children’s behaviors in intergroup contexts (Nesdale et al., 2007, 2010; Watson-Jones et al., 2016). In one experiment (Nesdale et al., 2010), 7- and 9-year-old children were asked to imagine that they were going to participate in an intergroup drawing competition that would involve children from other schools in the area. Next, the children in the inclusion condition were informed that their team members liked the participant’s drawing and had explicitly asked the participant to join the team. In contrast, the children in the exclusion condition were informed that their team members did not like the participant’s drawing and that they did not want the participant on the team. More relevant to the present study, children who received feedback signaling ingroup inclusion displayed more positive attitudes toward the members of their outgroup (e.g., how much they like, trust, and would want to play with the members) than children who received feedback signaling ingroup exclusion.

The present study focused on prosocial behaviors in children, primarily sharing, and investigated the impact of ingroup inclusion and exclusion on ingroup favoritism in 6-year-old children. Developmental research has indicated that children show ingroup favoritism in sharing behaviors (Zinser et al., 1981; Kinzler et al., 2007; Fehr et al., 2008; Gummerum et al., 2009; Moore, 2009; Paulus and Moore, 2014; Yu et al., 2016; Sparks et al., 2017; Yazdi et al., 2020). These results were mainly obtained *via* three different types of tasks. First, in resource-allocation tasks, children are typically asked to distribute scarce resources to ingroup and outgroup individuals but are not allowed to reserve any resources for themselves. In these mixed-recipient resource allocation tasks, preschool children allocate more resources to ingroup versus outgroup members (Olson and Spelke, 2008; Dunham et al., 2011; Renno and Shutts, 2015). For example, Olson and Spelke (2008) reported that 3-year-old children directed a protagonist puppet to give more resources to the protagonist’s friends or siblings than to strangers.

Second, in forced-choice sharing tasks (i.e., mini-dictator games), children are asked to choose a desirable outcome between two resource allocation options involving themselves and a partner. In these scenarios, children are more likely to share their own resources when their partner is a member of their ingroup versus a member of their outgroup (Fehr et al., 2008; Moore, 2009; Paulus and Moore, 2014; Yu et al., 2016; Sparks et al., 2017). For example, Fehr et al. (2008) asked children to choose how sweets should be shared between themselves and an ingroup partner (an anonymous child from the same school) or an outgroup partner (an anonymous child from a different school). Specifically, the children were asked to choose between an allocation of one sweet for themselves and one sweet for their partner (1,1) and an allocation of (2,0). The researchers found that children aged 7–8 were more likely to choose the equal (1,1) allocation when their partner was an ingroup member compared to when they were an outgroup member. Using a similar paradigm, Yu et al. (2016) found that 5- to 6-year-old children showed ingroup favoritism in that they were more likely to choose an equal sharing option when their partner was an ingroup member versus an outgroup member.

Finally, in the third type of sharing task, known as the dictator game, children freely chose how many (if any) of a set number of items to allocate between themselves and their partner. Compared with the others tasks, the dictator game is less frequently used to examine costly sharing in children in relation to group membership. This is likely due in part to difficulties in curbing self-interest during dictator games. However, children have been found to share more resources with individuals in their ingroup versus outgroup in the dictator game, and this pattern becomes clearer with age (Gummerum et al., 2009; Benozio and Diesendruck, 2015; McLoughlin and Over, 2019; Yazdi et al., 2020). For example, Gummerum et al. (2009) asked 7- and 11-year-old children to play a dictator game using money, and found that older but not younger children allocated significantly more resources to members of their own group. Related to this finding, Yazdi et al. (2020) asked 5- to 9-year-old children to allocate 10 stickers between themselves and an ingroup member or an outgroup member either in the presence of an adult observer or alone, and found that children shared more resources with the ingroup member regardless of the existence of the observer. Studies with younger children revealed more individual variance, for instance, in children 3 to 5 years of age, only boys showed ingroup favoritism in a dictator game where they were allowed to allocate 10 stickers between themselves and an ingroup or outgroup partner (Benozio and Diesendruck, 2015).

Taken together, the above results suggest that, at least in certain situations, children selectively share more with their ingroup members than with outgroup members. In the present study, we explored how children’s ingroup favoritism in terms of sharing behaviors are influenced by their previousinteractions with ingroup members. Specifically, we investigated the impact of the experience of being included or excluded by an ingroup on children’s costly sharing with ingroup versus outgroup members in dictator games. In the present study, children were first assigned to one of two minimal groups marked by different colors (e.g., Dunham et al., 2011). Next, children in two conditions, *ingroup-inclusion* and *ingroup-exclusion*, played a brief Cyberball game (Williams and Jarvis, 2006). Cyberball is a virtual ball-tossing game that has previously been used to manipulate the experience of inclusion or exclusion by group members (Watson-Jones et al., 2016). Following this manipulation, we measured the way that the children shared with ingroup and outgroup members (i.e., how many stickers they chose to share). We used the dictator game because we hoped to provide stronger evidence regarding prosocial tendencies in children, as well as to contribute to the sparse literature on young children’s ingroup favoritism in dictator games. We predicted that 6-year-old children would exhibit less ingroup favoritism, as indicated by their selective sharing, with ingroup versus outgroup members when they were included by the ingroup compared to when they were excluded. We also included one more condition, a *no-interaction* condition in which the children did not play Cyberball. The inclusion of this condition helped address directionality of the effect, i.e., does ingroup inclusion reduce ingroup favoritism or does ingroup exclusion promote it, or both?

## Experiment

### Method

#### Participants

Ninety 6-year-old Korean children (72.0–84.3 months, M = 77.34, SD = 3.75, 44 girls) participated in the experiment. An additional five children participated but were excluded because they were too active or fussy (3), or because of parental interference (2). Thirty children were randomly assigned to one of three conditions (ingroup-inclusion, ingroup-exclusion, no-interaction condition). We conducted an *a priori* power analysis (G*Power 3.1; Faul et al., 2007) for a 3 (condition) × 2 (group) analysis of variance (ANOVA). Based on the effect size of the previous research on the similar topic (McLoughlin and Over, 2019), for a power of 0.80 and with an α of 0.05, a minimum of 78 participants were required. Nevertheless, we included 30 participants in each condition (total of 90 participants). The children were given a book to thank them for their participation. Each child’s parent provided written informed consent, and the study protocol was approved by the Institutional Review Board of Sungshin Women’s University.

#### Apparatus

This experiment was conducted during the COVID-19 pandemic, and so the children participated using an online system. The visual stimuli were created using Microsoft PowerPoint (Microsoft Corp., Redmond, WA, United States). During the experiment, an experimenter interacted with each child online. Visual stimuli were presented to the children using the “screen sharing” function in Zoom (Zoom Video Communications, Inc., San Jose, CA, United States). Prior to the study, parents were given instructions as to how to set up their screen (a single monitor of a specific size, Zoom video settings, etc.), a recording tool (centered webcam, etc.), sound (the computer volume), and the environment (faces clearly visible, minimizing distractions, etc.). We recorded the shared screen during the session. To prevent any interference during the experiment, the parents were instructed to leave the room.

#### Materials and procedure

##### Ingroup conditions (Ingroup-inclusion, Ingroup-exclusion)

The experiment consisted of three phases: group-allocation, Cyberball, and sharing. The children were tested individually by an experimenter using Zoom. The entire experiment took approximately 15 min to complete.

###### Group allocation

In the group allocation phase, the children were assigned to one of two groups (a yellow or a green group) in a minimal-group paradigm (Tajfel et al., 1971). The children were first presented with three slides that each showed a pair of objects belonging to the same category, such as pet animals (cat vs. dog), fruit (apple vs. pear), and playground equipment (swings vs. slide), and were asked which one they liked better. On the next slide, each pair of objects appeared in a row, and the child’s preferred choices were marked with a red circle. The experimenter reminded the children of their choices by saying, “So you said you like cats, apples, and swings better,” while pointing at the child’s preferred choices using a mouse cursor. The experimenter then introduced two groups, the yellow and the green group, one on each side of the screen, and showed two illustrated characters wearing yellow T-shirts and two characters wearing green T-shirts. The gender of the characters matched the child’s gender. Then, the children were told that, depending on their preferred choices, they would be assigned to the yellow or green group, e.g., “Children in the yellow (or green) group like cats, apples, and swings, just like you. So now you are in the yellow group.” The experimenter then asked the child to which color group they belonged. All of the children who participated in the present experiment correctly identified their group membership.

###### Cyberball

**Figure 1:**
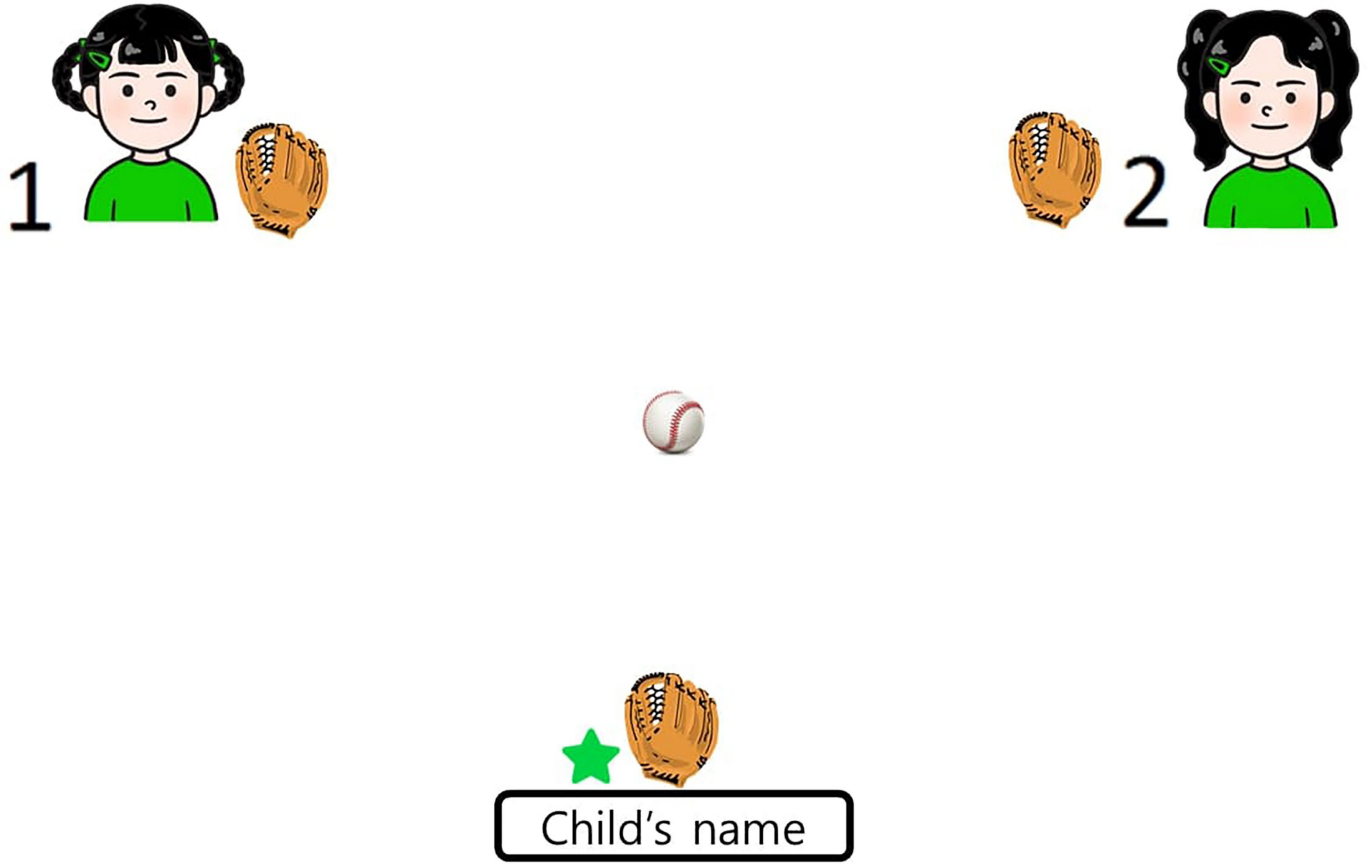
Example of Cyberball phase. In the Ingroup-inclusion condition, children received 5 ball tosses out of a total of 15 tosses, while in the Ingroup-exclusion conditions they received only 1 ball toss.

Next, in the Cyberball phase, children in the ingroup-inclusion and ingroup-exclusion conditions were told that the computer would connect them with their group members so that they could play a ball-tossing game together. We adapted the age appropriate Cyberball paradigm for children (Abrams et al., 2011; Watson-Jones et al., 2016; Hwang and Markson, 2020). Children saw two members of their ingroup, i.e., the illustrated characters, one on the left side and the other on the right side of the upper screen, each with a baseball glove (Figure 1). At the lower center area of the screen, an illustrated baseball glove with the name of the participant was shown with a colored star. To increase the saliency of group membership, the color of the star on the baseball glove matched the color of the child’s group. At the beginning of the game, a ball appeared in the glove of one of the other players. The participants could only toss the ball to the other players when the ball was in their glove. Since the present study was conducted online, the two ingroup members were number-coded (1 and 2). To toss the ball, the children had to read the number that corresponded to the player to which they wanted to toss the ball. The experimenter explained how to play the game by saying, “This is a ball tossing game. When you have the ball, it’s your turn to throw the ball. If you want to throw the ball to this child (while pointing at the character on the left, which was numbered as (1), you say “One!” and the ball will be thrown to that child. When you want to throw the ball to this child (while pointing at the character on the right, numbered as (2), you say “Two!” and the ball will be thrown to that child. You can choose to pass the ball to any player you want and the other players will choose to whom they are going to pass the ball. While you are playing the game, I want you to imagine that you are in the playground, actually passing a ball back and forth with the other players in the game. Okay?” After completing a practice game, the children played the Cyberball game. The two ingroup conditions differed in terms of how many ball tosses the children received from the ingroup members. The children in the ingroup-inclusion condition received 5 ball tosses out of a total of 15 tosses; whereas the children in the ingroup-exclusion condition received only 1 ball toss and they were left out of the game for the remaining ~2 min of game play.

Following the Cyberball game, we performed a *manipulation check* to ensure that the children recognized the situation of inclusion versus exclusion. The experimenter asked the children, “How much did they throw you the ball? A lot or a little?” while presenting thumbs-up and thumbs-down signs side-by-side on the screen. Next, we asked the children a *moral-evaluation question* to explore how they evaluated the ingroup individuals who either included or excluded them. The experimenter asked the children, “What do you think about the children who played the ball-tossing game with you? Were they very bad, bad, not bad nor nice, nice, or very nice?” while showing a 5-point scale with stars representing positive ratings (from two stars representing very nice to one star representing nice), X representing negative ratings (from XX representing very bad to X representing bad), and a blank circle representing not nice nor bad. The ratings were coded from 1 (very bad) to 5 (very nice). To check for differences in emotional experience following the inclusion and exclusion games, we also asked children how they felt after they played the ball-tossing game. For *emotion ratings*, the experimenter asked, “How did you feel during the ball tossing game?” while showing a 5-point scale with representative drawings of a face depicting the relevant emotion. The ratings were then coded from 1 (very bad) to 5 (very good).

###### Sharing

Next, in the sharing phase, the children played two rounds of the dictator game in the role of the proposer, in which they were asked to divide their stickers between themselves and either an ingroup or an outgroup member (order was counterbalanced across the children). In each trial, the children were presented with two boxes: one on the top and one on the bottom center area of the screen. The children were told that the bottom box belonged to them and that the top box belonged to another child who was either an ingroup or outgroup member. To help the children understand the task, their name was written next to their box, and the face of the illustrated character (the ingroup or outgroup member) was presented next to his or her box. The ingroup member was one of the other players in the Cyberball game, and the outgroup member was one of the two outgroup members who were previously introduced in the group-allocation phase. Next, the experimenter presented five identical red heart-shaped ‘stickers’ on the screen and told the participant that they could give some of their stickers to their partner if they wanted, or they could keep the stickers: “Look, here are your stickers. Do you want to count them with me?” After pausing to let the child count the stickers, the experimenter said, “Right, you have five stickers! Look, this is your box,” and pointed to the bottom box. The experimenter then said, “I put your name on this box so you know that this is yours. And look, this box is for this yellow (or green) group child,” while pointing to the top box. Then, the experimenter told the children that they could share their stickers: “If you want to, you can keep your stickers. Or, if you want to, you can give your stickers to this child from the yellow (or green) group, as many as you want.” To make the online experiment more realistic, the experimenter informed the children that she would mail the actual stickers to the participant’s home right after the experiment. The children were asked to indicate verbally whether they wanted to share the stickers and, if they wanted to share, how many stickers they wanted to give away. The experimenter moved the stickers next to the recipient’s box following the children’s responses. The number of stickers that the participant shared with the other child served as the main dependent variable of the present experiment. After the experiment was complete, the children were informed that the players in the Cyberball game were not real people, and the children in the ingroup-exclusion condition were allowed to play the inclusion game to alleviate any negative emotions caused by the exclusion game.

##### No-interaction condition

The materials and procedure in the no-interaction condition were similar to those in the ingroup-exclusion and ingroup-inclusion conditions except for a key difference: the children in the no-interaction condition did not complete the Cyberball phase. Therefore, the no-interaction condition consisted of two phases, the group-allocation and the sharing phase. Following the group-allocation phase, the children completed an emotion rating task in which we asked them about their current emotions, and then completed the sharing task. The full experiment took approximately 10 min to complete.

## Results

### Manipulation check

In the ingroup-inclusion condition, 27/30 (90.0%) children responded that they received many ball tosses during the ball-tossing game, *p* < 0.001 (cumulative binomial probability). Meanwhile, in the ingroup-exclusion condition, 27/30 (90.0%) children responded that they received few ball tosses during the ball-tossing game, *p* < 0.001. The distributions of the binary choices (many vs. few) in the two conditions were significantly different according to a 2 × 2 Fisher’s exact test, *p* < 0.001.

### Moral evaluation

We conducted an independent samples *t*-test with condition (ingroup-inclusion or ingroup-exclusion) as a between-subjects factor to determine whether the children in the two conditions gave different evaluations for their ingroup members with whom they played Cyberball. Children in the ingroup-inclusion condition (M = 4.23, SD = 1.04) evaluated their ingroup members more positively than those in the ingroup-exclusion condition (M = 3.07, SD = 1.34), *t*(58) = 3.772, *p* < 0.001. In addition, we performed one sample *t*-tests to compare the moral evaluations of ingroup members against the midpoint (3, not bad nor nice). Children in the ingroup-inclusion condition evaluated their ingroup members as nice, *t*(29) = 6.495, *p* < 0.001, whereas the average evaluation for ingroup members in the ingroup-exclusion condition was not reliably different from the midpoint, *t*(29) = 0.273, *p* = 0.787.

### Emotion ratings

An independent samples *t*-test with condition (ingroup-inclusion or ingroup-exclusion) as a between-subjects factor revealed that the children in the ingroup-inclusion condition (M = 4.33, SD = 0.76) felt more positive emotions than the children in the ingroup-exclusion condition (M = 3.67, SD = 1.12), *t*(58) = 2.693, *p* = 0.009. One sample *t*-tests revealed that in both conditions, the emotional ratings were significantly above the midpoint (3, not bad nor good; ingroup-inclusion condition: *t*(29) = 9.633, *p* < 0.001, ingroup-exclusion condition: *t*(29) = 3.247, *p* = 0.003). This indicates that the Cyberball paradigm is safe to use with young children because the children in the exclusion condition reported their emotions as slightly positive, i.e., better than neutral. The emotional ratings in the no-interaction condition (M = 4.00, SD = 0.95) were not significantly different to those of the ingroup-inclusion condition, *t*(58) = −1.505, *p* = 0.138, and those of the ingroup-exclusion condition, *t*(58) = 1.242, *p* = 0.219.

### Sharing

**Figure 2:**
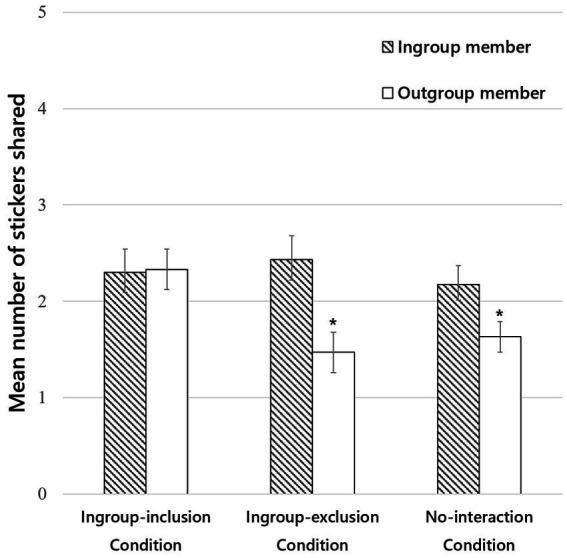
Mean number of stickers children shared with the ingroup or outgroup member. Error bars represent standard error.

Preliminary analyses of the test data revealed no interaction of condition and recipient group with children’s sex or test order, all *F*s(2, 84) < 0.687, *p*s > 0.506. Therefore, the data were collapsed across the latter two factors. Sharing behavior (Figure 2) was subjected to an ANOVA with condition (ingroup-inclusion, ingroup-exclusion, no-interaction) as a between-subjects factor and recipient’s group (ingroup, outgroup) as a within-subject factor. The analysis yielded no significant main effect of condition, *F*(2, 87) = 1.810, *p* = 0.170, η_p_^2^ = 0.04, a significant main effect of recipient’s group, *F*(1, 87) = 10.670, *p* = 0.002, η_p_^2^ = 0.11, and, crucially, a significant condition × group interaction, *F*(2, 87) = 3.742, *p* = 0.028, η_p_^2^ = 0.08. Children in the ingroup-inclusion condition shared equally with the ingroup (M = 2.30, SD = 1.29) and outgroup members (M = 2.33, SD = 1.16), *t*(29) = −0.114, *p* = 0.910. However, children in the ingroup-exclusion condition shared more stickers with the ingroup (M = 2.43, SD = 1.36) versus the outgroup member (M = 1.47, SD = 1.17), *t*(29) = 3.846, *p* = 0.001. Similarly, children in the no-interaction condition shared more stickers with the ingroup (M = 2.17, SD = 1.09) versus the outgroup member (M = 1.63, SD = 0.89), *t*(29) = 2.333, *p* = 0.027.

## Discussion

The present study investigated whether a sense of ingroup belonging decreases children’s ingroup favoritism in costly sharing behavior. Six-year-old children shared more resources with a member of their minimal ingroup versus an outgroup member, both when they were previously excluded by the ingroup or when they had no particular interaction with the ingroup. In contrast, they shared equally with the ingroup and outgroup members when they were clearly included by the ingroup. These results suggest that ingroup inclusion may reduce children’s ingroup favoritism.

The present results indicate that belonging to the group is one of the important motives underlying children’s selective prosociality toward the ingroup. It is well documented that young children preferentially allocate resources toward ingroup members compared with outgroup members (e.g., Fehr et al., 2008; Olson and Spelke, 2008; Dunham et al., 2011; Kinzler et al., 2012; Benozio and Diesendruck, 2015; Renno and Shutts, 2015; Yu et al., 2016). Moreover, by the end of the second year of life, even young infants hold expectations about ingroup care: infants expect individuals to help in-group members in need (Jin and Baillargeon, 2017) and to reserve scarce resources for ingroup members as opposed to outgroup members (Bian et al., 2018). While very informative, this prior work leaves questions regarding the social circumstances under which young children show ingroup favoritism and the motivation for these behaviors. Our experimental approach using the Cyberball paradigm allowed us to address these questions. We found that being excluded by the ingroup, even in a brief interaction, or having no clear indication regarding ingroup membership led children to be more willing to share in favor of the ingroup. By contrast, children included by the ingroup were more willing to share equally with the ingroup and outgroup. Thus, this study represents the first evidence that belonging to a group reduces children’s tendency to favor their minimal ingroup over the outgroup.

Notably, the present results suggest that inclusion status within a group can modulate intergroup bias. The vast majority of intergroup bias interventions focused on changing children’s representations of, and emotional responses to, outgroup members (Cameron et al., 2006; Cameron and Rutland, 2006; Aboud et al., 2012; Vezzali et al., 2012, 2015; Sierksma et al., 2014, 2015; Abrams et al., 2015; McLoughlin and Over, 2019). However, implementing these interventions in reality can be challenging and may actually intensify intergroup bias in adults (Dixon et al., 2007; Dovidio et al., 2009; Vorauer and Sasaki, 2009). The experimental manipulation in the present study was designed to enhance children’s ingroup belonging while overcoming these challenges. In children, clear acceptance by a group through positive interactions with its members may decrease preferential treatment of ingroup over outgroup members. Understanding the link between ingroup belonging and ingroup favoritism is important for effective and affordable interventions (Walton and Wilson, 2018) and educational programs to reduce the negative consequences of ingroup favoritism, including outgroup derogation and prejudice.

Our findings are consistent with theoretical insights derived from adult social psychology (e.g., Brewer, 1991; Baumeister and Leary, 1995; Branscombe et al., 1999; Fiske, 2004; Leary, 2005). Baumeister and Leary (1995) viewed the need to belong as a fundamental motivation for the formation of long-lasting interpersonal relationships. Other researchers have elaborated further on this need to belong to groups (Brewer, 1991; Fiske, 2004). For example, from the perspective of optimal distinctiveness theory (Brewer, 1991), Leonardelli et al. (2010) argued that individuals might exhibit ingroup favoritism to meet their need for inclusion in an effort to gain acceptance or inclusion by the group. The optimal distinctiveness theory posits that humans have two fundamental needs, the need for group inclusion and the need for a sense of uniqueness, and the conflict between the two needs is resolved through achieving an optimal level of distinctiveness. In this regard, our tendency to treat ingroup members more positively than outgroup members might be a way to maintain intergroup differences such that they are greater than intragroup differences. A sense of ingroup inclusion could reduce such ingroup bias because it would encourage group members to focus on ingroup similarities as opposed to intergroup differences.

It is interesting to note that the children in the ingroup-exclusion and no-interaction conditions showed similar behaviors in that they shared more resources with the ingroup versus outgroup members, whereas children in the ingroup-inclusion condition shared equally with the ingroup and outgroup members. We speculate that children show relatively pronounced ingroup favoritism unless they have a clear indication that they are included by their ingroup (e.g., Leonardelli et al., 2010). For instance, in the no-interaction condition, children were assigned to a new minimal group, and the only information they knew about the group members was that they had the same preferences for some objects. That is, children in the no-interaction condition were not given a firm guarantee that they would be welcomed and included by their ingroup members in future interactions. For this reason, children in the no-interaction condition might have shown the same level of ingroup favoritism as those in the ingroup-exclusion condition.

This was the first study to document ingroup favoritism among children, in the context of costly sharing, using the minimal-group paradigm (MGP) in the setting of a collective culture. Prior research on children’s ingroup favoritism using the MGP was conducted almost exclusively within Western cultures (Patterson and Bigler, 2006; Dunham et al., 2011; Rhodes, 2012; Plötner et al., 2015; Misch et al., 2016; Richter et al., 2016; Sparks et al., 2017; Dunham, 2018; Over et al., 2018; Yazdi et al., 2020; see for exceptions, Lee et al., 2018; Yang et al., 2021; Yang and Park, 2022), and their findings may not be applicable to other cultures. Although ingroup favoritism has been suggested as an innate human tendency (Tajfel et al., 1971; Brewer, 1979), the level of ingroup favoritism may differ across cultures (Fischer and Derham, 2016). Interestingly, an association between individualism–collectivism and ingroup bias in the MGP has been observed in adults in both directions. On one hand, collectivism may be associated with higher in-group bias in the MGP because individuals in collectivistic cultures tend to value harmony among group members and cooperation to achieve mutual goals (Triandis, 1990, 1994). Moreover, the social enjoinments are characterized by close-knit groups (Yamagishi et al., 1998). On the other hand, collectivism may also be associated with lower in-group bias in the MGP because individuals in collectivistic cultures already have strong, stable, and immutable individual-group associations, and are not concerned about their identities or inclusion in novel minimal groups (Hogg, 2000, 2007; Falk et al., 2014). Given the multiple possibilities, cross-cultural research on the development of ingroup favoritism in MGPs across age groups may be particularly informative. We found that 6-year-old Korean children displayed minimal group effects in the context of resource allocation, similar to results from Western studies (Dunham et al., 2011; Sparks et al., 2017). This cross-culture consistency indicates that ingroup favoritism in the MGP is culturally consensual, at least around the age of 6 years. However, further large-scale studies are required to examine the minimal group effects in on-Western cultures in different age groups.

The present results contribute to literature on the impact of social exclusion on social behavior in children. Previous research has shown that young children can correctly identify social exclusion and that they prefer inclusive agents to exclusive agents (Hwang and Markson, 2020). As they get older, children are increasingly able to make moral decisions about social exclusion (Killen and Rutland, 2011; Will et al., 2013). Furthermore, a brief experience of social exclusion was found to increase imitation behaviors in children (Over, 2016), including imitative fidelity (Over and Carpenter, 2009; Watson-Jones et al., 2014, 2016), nonconscious facial mimicry (de Klerk et al., 2020), and imitation in referential and syntactic choices (Hopkins and Branigan, 2020). The present findings provide evidence that ingroup exclusion and inclusion affect a different type of social behavior, i.e., the tendency of children to favor ingroup members during sharing. These results suggest that the need to belong is an important driver of sociomoral development in children.

One interesting topic for future research is the way in which reputation concerns amongst children might impact ingroup favoritism after they experience ingroup inclusion or exclusion. Adults whose sense of belonging to their ingroup was threatened made greater distinctions between the ingroup and outgroup, especially when they were in public as opposed to a private setting (Noel et al., 1995). These results suggest that adults are able to exploit ingroup favoritism as a means by which to display their willingness to ingratiate themselves to a group. Relatedly, previous research has suggested that young children are also motivated by a desire to make a positive impression on others (Piazza et al., 2011; Engelmann et al., 2012, 2013; Fu et al., 2012, 2016; Leimgruber et al., 2012; Zhao et al., 2017; Rapp et al., 2019; Yazdi et al., 2020). For example, 5-year-old children are more likely to share when they are in the presence of a peer than when they are alone (Engelmann et al., 2012). If children use ingroup favoritism as a means by which to affiliate with ingroup members, children who seek to belong to ingroups would be expected to become more preferential toward ingroups when they are in public versus private contexts. In addition, it will be interesting to investigate whether the effects of inclusion and exclusion arise only from ingroup interactions, or also from other types of social interactions (e.g., with individuals whose group membership is unknown or outgroup individuals). Future studies that test these possibilities are needed to further our understanding of the motivations underlying human ingroup favoritism.

## Conclusion

The present study is the first to demonstrate that ingroup belonging may reduce children’s ingroup favoritism in terms of cost sharing. In a minimal-group paradigm, 6-year-old children shared more of their resources with their ingroup members than outgroup members. This was the case both when they were excluded by their ingroup members and when they had no particular history of interaction with them. In contrast, children did not show such ingroup favoritism when they were included by their ingroup members. This new finding suggests that children are sensitive to information about ingroup belonging and will respond accordingly.

## Data availability statement

The raw data supporting the conclusions of this article will be made available by the authors, without undue reservation.

## Ethics statement

The studies involving human participants were reviewed and approved by Internal Review Board of Sungshin Women’s University. Written informed consent to participate in this study was provided by the participants’ legal guardian/next of kin.

## Author contributions

JP and KJ developed the study concept and design. Data were collected and analyzed by JP and KJ. JP wrote the first draft. KJ made critical revisions. All authors contributed to the article and approved the submitted version.

## Funding

This work was supported by the National Research Foundation of Korea (NRF) grant funded by the Korea government (MSIT) (No. 2020R1G1A1014507) to KJ.

## Conflict of interest

The authors declare that the research was conducted in the absence of any commercial or financial relationships that could be construed as a potential conflict of interest.

## Publisher’s note

All claims expressed in this article are solely those of the authors and do not necessarily represent those of their affiliated organizations, or those of the publisher, the editors and the reviewers. Any product that may be evaluated in this article, or claim that may be made by its manufacturer, is not guaranteed or endorsed by the publisher.

## References

[ref1] Aboud F. E. Tredoux C. Tropp L. R. Brown C. S. Niens U. Noor N. M. (2012). Interventions to reduce prejudice and enhance inclusion and respect for ethnic differences in early childhood: a systematic review. Dev. Rev. 32, 307–336. doi: 10.1016/j.dr.2012.05.001

[ref2] Abrams D. Van de Vyver J. Pelletier J. Cameron L. (2015). Children's prosocial behavioural intentions towards outgroup members. Br. J. Dev. Psychol. 33, 277–294. doi: 10.1111/bjdp.12085, PMID: 25773274

[ref3] Abrams D. Weick M. Thomas D. Colbe H. Franklin K. M. (2011). On-line ostracism affects children differently from adolescents and adults. Br. J. Dev. Psychol. 29, 110–123. doi: 10.1348/026151010X494089, PMID: 21288256

[ref4] Baillargeon R. Scott R. M. He Z. Sloane S. Setoh P. Jin K.. (2015). “Psychological and Sociomoral reasoning in infancy,” in APA handbook of personality and social psychology. eds. Mikulincer M. Shaver P. R. Borgida E. Bargn J. A., Attitudes and social cognition. vol. 1 (Washington, DC: American Psychological Association), 79–150.

[ref5] Balliet D. Wu J. De Dreu C. K. (2014). Ingroup favoritism in cooperation: a meta-analysis. Psychol. Bull. 140, 1556–1581. doi: 10.1037/a0037737, PMID: 25222635

[ref6] Baumeister R. F. Leary M. R. (1995). The need to belong: desire for interpersonal attachments as a fundamental human motivation. Psychol. Bull. 117, 497–529. doi: 10.1037/0033-2909.117.3.497, PMID: 7777651

[ref7] Benozio A. Diesendruck G. (2015). Parochialism in preschool boys’ resource allocation. Evol. Hum. Behav. 36, 256–264. doi: 10.1016/j.evolhumbehav.2014.12.002

[ref8] Bian L. Sloane S. Baillargeon R. (2018). Infants expect ingroup support to override fairness when resources are limited. Proc. Natl. Acad. Sci. U. S. A. 115, 2705–2710. doi: 10.1073/pnas.1719445115, PMID: 29483252 PMC5856544

[ref9] Branscombe N. R. Ellemers N. Spears R. Doosje B. (1999). “The context and content of social identity threat” in Social identity: Context, commitment, content. eds. Ellemers N. Spears R. Doosje B. (Oxford, UK: Blackwell Science), 35–58.

[ref10] Brewer M. B. (1979). In-group bias in the minimal intergroup situation: a cognitive-motivational analysis. Psychol. Bull. 86, 307–324. doi: 10.1037/0033-2909.86.2.307

[ref11] Brewer M. B. (1991). The social self: on being the same and different at the same time. Personal. Soc. Psychol. Bull. 17, 475–482. doi: 10.1177/0146167291175001

[ref12] Brewer M. B. (1999). The psychology of prejudice: Ingroup love and outgroup hate? J. Soc. Issues 55, 429–444. doi: 10.1111/0022-4537.00126

[ref13] Brown G. T. Peterson E. R. Yao E. S. (2016). Student conceptions of feedback: impact on self-regulation, self-efficacy, and academic achievement. Br. J. Educ. Psychol. 86, 606–629. doi: 10.1111/bjep.12126, PMID: 27612004

[ref14] Cameron L. Rutland A. (2006). Extended contact through story reading in school: reducing children's prejudice toward the disabled. J. Soc. Issues 62, 469–488. doi: 10.1111/j.1540-4560.2006.00469.x

[ref15] Cameron L. Rutland A. Brown R. Douch R. (2006). Changing children's intergroup attitudes toward refugees: testing different models of extended contact. Child Dev. 77, 1208–1219. doi: 10.1111/j.1467-8624.2006.00929.x, PMID: 16999793

[ref16] de Klerk C. Albiston H. Bulgarelli C. Southgate V. Hamilton A. (2020). Observing third-party ostracism enhances facial mimicry in 30-month-olds. J. Exp. Child Psychol. 196:104862. doi: 10.1016/j.jecp.2020.104862, PMID: 32353814 PMC7262587

[ref17] Dixon J. Durrheim K. Tredoux C. (2007). Intergroup contact and attitudes toward the principle and practice of racial equality. Psychol. Sci. 18, 867–872. doi: 10.1111/j.1467-9280.2007.01993.x, PMID: 17894603

[ref18] Dovidio J. F. Gaertner S. L. Saguy T. (2009). Commonality and the complexity of “we”: social attitudes and social change. Personal. Soc. Psychol. Rev. 13, 3–20. doi: 10.1177/1088868308326751, PMID: 19144903

[ref19] Dunham Y. (2018). Mere membership. Trends Cogn. Sci. 22, 780–793. doi: 10.1016/j.tics.2018.06.004, PMID: 30119749

[ref20] Dunham Y. Baron A. S. Carey S. (2011). Consequences of “minimal” group affiliations in children. Child Dev. 82, 793–811. doi: 10.1111/j.1467-8624.2011.01577.x, PMID: 21413937 PMC3513287

[ref21] Engelmann J. M. Herrmann E. Tomasello M. (2012). Five-year olds, but not chimpanzees, attempt to manage their reputations. PLoS One 7:e48433. doi: 10.1371/journal.pone.0048433, PMID: 23119015 PMC3485200

[ref22] Engelmann J. M. Over H. Herrmann E. Tomasello M. (2013). Young children care more about their reputation with ingroup members and potential reciprocators. Dev. Sci. 16, 952–958. doi: 10.1111/desc.12086, PMID: 24118719

[ref23] Falk C. F. Heine S. J. Takemura K. (2014). Cultural variation in the minimal group effect. J. Cross-Cult. Psychol. 45, 265–281. doi: 10.1177/0022022113492892

[ref24] Faul F. Erdfelder E. Lang A. Buchner A. (2007). G* power 3: a flexible statistical power analysis program for the social, behavioral, and biomedical sciences. Behav. Res. Methods 39, 175–191. doi: 10.3758/BF03193146, PMID: 17695343

[ref25] Fehr E. Bernhard H. Rockenbach B. (2008). Egalitarianism in young children. Nature 454, 1079–1083. doi: 10.1038/nature07155, PMID: 18756249

[ref26] Fischer R. Derham C. (2016). Is in-group bias culture-dependent? A meta-analysis across 18 societies. Springer Plus 5, 70–79. doi: 10.1186/s40064-015-1663-6, PMID: 26839763 PMC4723375

[ref27] Fiske S. T. (2004). Intent and ordinary bias: unintended thought and social motivation create casual prejudice. Soc. Justice Res 17, 117–127. doi: 10.1023/B:SORE.0000027405.94966.23

[ref28] Fiske S. T. (2010). Social beings: Core motives in social psychology. New York: Wiley.

[ref29] Fu G. Evans A. D. Xu F. Lee K. (2012). Young children can tell strategic lies after committing a transgression. J. Exp. Child Psychol. 113, 147–158. doi: 10.1016/j.jecp.2012.04.003, PMID: 22704035

[ref30] Fu G. Heyman G. D. Qian M. Guo T. Lee K. (2016). Young children with a positive reputation to maintain are less likely to cheat. Dev. Sci. 19, 275–283. doi: 10.1111/desc.12304, PMID: 25872952

[ref31] Gómez Á. Morales J. F. Hart S. Vázquez A. Swann W. B. Jr. (2011). Rejected and excluded forevermore, but even more devoted: irrevocable ostracism intensifies loyalty to the group among identity-fused persons. Personal. Soc. Psychol. Bull. 37, 1574–1586. doi: 10.1177/0146167211424580, PMID: 22045779

[ref32] Graham J. Haidt J. Koleva S. Motyl M. Iyer R. Wojcik S. P.. (2013). “Moral foundations theory: the pragmatic validity of moral pluralism” in Advances in experimental social psychology. eds. Devine P. Plant A. (San Diego, CA: Academic Press), 55–130.

[ref33] Greitemeyer T. (2012). Boosting one's social identity: effects of social exclusion on ethnocentrism. Basic Appl. Soc. Psychol. 34, 410–416. doi: 10.1080/01973533.2012.712013

[ref34] Gummerum M. Takezawa M. Keller M. (2009). The influence of social category and reciprocity on adults' and children's altruistic behavior. Evol. Psychol. 7, 147470490900700–147470490900316. doi: 10.1177/147470490900700212

[ref35] Hewstone M. Rubin M. Willis H. (2002). Intergroup bias. Annu. Rev. Psychol. 53, 575–604. doi: 10.1146/annurev.psych.53.100901.135109 11752497

[ref36] Hogg M. A. (2000). Subjective uncertainty reduction through self-categorization: a motivational theory of social identity processes. Eur. Rev. Soc. Psychol. 11, 223–255. doi: 10.1080/14792772043000040

[ref37] Hogg M. A. (2007). Uncertainty–identity theory. Adv. Exp. Soc. Psychol. 39, 69–126. doi: 10.1016/S0065-2601(06)39002-8

[ref38] Hopkins Z. L. Branigan H. P. (2020). Children show selectively increased language imitation after experiencing ostracism. Dev. Psychol. 56, 897–911. doi: 10.1037/dev0000915, PMID: 32191052 PMC7144459

[ref39] Hwang H. G. Markson L. (2020). The development of social exclusion detection in early childhood: awareness of social exclusion does not always align with social preferences. J. Cogn. Dev. 21, 166–190. doi: 10.1080/15248372.2019.1706521, PMID: 36017111 PMC9401208

[ref40] Jetten J. Postmes T. McAuliffe B. J. (2002). ‘We're all individuals’: group norms of individualism and collectivism, levels of identification and identity threat. Eur. J. Soc. Psychol. 32, 189–207. doi: 10.1002/ejsp.65

[ref41] Jin K. Baillargeon R. (2017). Infants possess an abstract expectation of ingroup support. Proc. Natl. Acad. Sci. U. S. A. 114, 8199–8204. doi: 10.1073/pnas.1706286114, PMID: 28716902 PMC5547641

[ref42] Killen M. Rutland A. (2011). Children and social exclusion: Morality, prejudice, and group identity. Oxford: John Wiley & Sons.

[ref43] Killen M. Turiel E. (1998). Adolescents' and young adults' evaluations of helping and sacrificing for others. J. Res. Adolesc. 8, 355–375. doi: 10.1207/s15327795jra0803_4

[ref44] Kinzler K. D. Dupoux E. Spelke E. S. (2007). The native language of social cognition. Proc. Natl. Acad. Sci. U. S. A. 104, 12577–12580. doi: 10.1073/pnas.0705345104, PMID: 17640881 PMC1941511

[ref45] Kinzler K. D. Dupoux E. Spelke E. S. (2012). ‘Native’ objects and collaborators: Infants' object choices and acts of giving reflect favor for native over foreign speakers. J. Cogn. Dev. 13, 67–81. doi: 10.1080/15248372.2011.567200, PMID: 23105918 PMC3478775

[ref46] Lakin J. L. Chartrand T. L. Arkin R. M. (2008). I am too just like you: nonconscious mimicry as an automatic behavioral response to social exclusion. Psychol. Sci. 19, 816–822. doi: 10.1111/j.1467-9280.2008.02162.x, PMID: 18816290

[ref47] Leary M. R. (2005). Sociometer theory and the pursuit of relational value: getting to the root of self-esteem. Eur. Rev. Soc. Psychol. 16, 75–111. doi: 10.1080/10463280540000007

[ref48] Leary M. R. Tambor E. S. Terdal S. K. Downs D. L. (1995). Self-esteem as an interpersonal monitor: the sociometer hypothesis. J. Pers. Soc. Psychol. 68, 518–530. doi: 10.1037/0022-3514.68.3.518

[ref1002] Lee K. J. J. Esposito, G. Setoh P. (2018). Preschoolers favor their Ingroup when resources are limited. Front. Psychol. 9:1752. doi: 10.3389/fpsyg.2018.01752 30283392 PMC6156258

[ref49] Leimgruber K. L. Shaw A. Santos L. R. Olson K. R. (2012). Young children are more generous when others are aware of their actions. PLoS One 7:e48292. doi: 10.1371/journal.pone.0048292, PMID: 23133582 PMC3485147

[ref50] Leonardelli G. J. Pickett C. L. Brewer M. B. (2010). “Optimal distinctiveness theory: a framework for social identity, social cognition, and intergroup relations” in Advances in experimental social psychology. eds. Zanna M. Olson J. (San Diego, CA: Academic Press), 63–113.

[ref51] Levine M. Prosser A. Evans D. Reicher S. (2005). Identity and emergency intervention: how social group membership and inclusiveness of group boundaries shape helping behavior. Personal. Soc. Psychol. Bull. 31, 443–453. doi: 10.1177/0146167204271651, PMID: 15743980

[ref52] McLoughlin N. Over H. (2019). Encouraging children to mentalise about a perceived outgroup increases prosocial behaviour towards outgroup members. Dev. Sci. 22, e12774–e12712. doi: 10.1111/desc.12774, PMID: 30451337

[ref53] Misch A. Over H. Carpenter M. (2016). I won’t tell: young children show loyalty to their group by keeping group secrets. J. Exp. Child Psychol. 142, 96–106. doi: 10.1016/j.jecp.2015.09.016, PMID: 26513328

[ref54] Moore C. (2009). Fairness in children's resource allocation depends on the recipient. Psychol. Sci. 20, 944–948. doi: 10.1111/j.1467-9280.2009.02378.x, PMID: 19515118

[ref55] Nesdale D. Durkin K. Maass A. Kiesner J. Griffiths J. Daly J.. (2010). Peer group rejection and children's outgroup prejudice. J. Appl. Dev. Psychol. 31, 134–144. doi: 10.1177/0165025407081479

[ref56] Nesdale D. Maass A. Kiesner J. Durkin K. Griffiths J. Ekberg A. (2007). Effects of peer group rejection, group membership, and group norms, on children's outgroup prejudice. Int. J. Behav. Dev. 31, 526–535. doi: 10.1016/j.appdev.2009.11.004

[ref57] Noel J. G. Wann D. L. Branscombe N. R. (1995). Peripheral ingroup membership status and public negativity toward outgroups. J. Pers. Soc. Psychol. 68, 127–137. doi: 10.1037/0022-3514.68.1.127, PMID: 7861310

[ref58] Olson K. R. Spelke E. S. (2008). Foundations of cooperation in young children. Cognition 108, 222–231. doi: 10.1016/j.cognition.2007.12.003, PMID: 18226808 PMC2481508

[ref59] Over H. (2016). The origins of belonging: social motivation in infants and young children. Philos. Trans. R. Soc. Lond. Ser. B Biol. Sci. 371:20150072. doi: 10.1098/rstb.2015.0072, PMID: 26644591 PMC4685518

[ref60] Over H. Carpenter M. (2009). Priming third-party ostracism increases affiliative imitation in children. Dev. Sci. 12, F1–F8. doi: 10.1111/j.1467-7687.2008.00820.x, PMID: 19371357

[ref61] Over H. Eggleston A. Bell J. Dunham Y. (2018). Young children seek out biased information about social groups. Dev. Sci. 21:e12580. doi: 10.1111/desc.12580, PMID: 28631413

[ref62] Patterson M. M. Bigler R. S. (2006). Preschool children's attention to environmental messages about groups: social categorization and the origins of intergroup bias. Child Dev. 77, 847–860. doi: 10.1111/j.1467-8624.2006.00906.x, PMID: 16942493

[ref63] Paulus M. Moore C. (2014). The development of recipient-dependent sharing behavior and sharing expectations in preschool children. Dev. Psychol. 50, 914–921. doi: 10.1037/a0034169, PMID: 23978297

[ref64] Piazza J. Bering J. M. Ingram G. (2011). “Princess Alice is watching you”: Children’s belief in an invisible person inhibits cheating. J. Exp. Child Psychol. 109, 311–320. doi: 10.1016/j.jecp.2011.02.003, PMID: 21377689

[ref65] Plötner M. Over H. Carpenter M. Tomasello M. (2015). The effects of collaboration and minimal-group membership on children’s prosocial behavior, liking, affiliation, and trust. J. Exp. Child Psychol. 139, 161–173. doi: 10.1016/j.jecp.2015.05.008, PMID: 26112747

[ref66] Powell L. J. Spelke E. S. (2013). Preverbal infants expect members of social groups to act alike. Proc. Natl. Acad. Sci. U. S. A. 110:E3965-E 3972. doi: 10.1073/pnas.1304326110, PMID: 24062446 PMC3799333

[ref67] Pun A. Birch S. A. Baron A. S. (2021). The power of allies: Infants' expectations of social obligations during intergroup conflict. Cognition 211:104630. doi: 10.1016/j.cognition.2021.104630, PMID: 33636572

[ref68] Rai T. S. Fiske A. P. (2011). Moral psychology is relationship regulation: moral motives for unity, hierarchy, equality, and proportionality. Psychol. Rev. 118, 57–75. doi: 10.1037/a0021867, PMID: 21244187

[ref69] Rapp D. J. Engelmann J. M. Herrmann E. Tomasello M. (2019). Young children’s reputational strategies in a peer group context. Dev. Psychol. 55, 329–336. doi: 10.1037/dev0000639, PMID: 30525833

[ref70] Renno M. P. Shutts K. (2015). Children’s social category-based giving and its correlates: expectations and preferences. Dev. Psychol. 51, 533–543. doi: 10.1037/a0038819, PMID: 25706588

[ref71] Rhodes M. (2012). Naïve theories of social groups. Child Dev. 83, 1900–1916. doi: 10.1111/j.1467-8624.2012.01835.x, PMID: 22906078

[ref72] Richter N. Over H. Dunham Y. (2016). The effects of minimal group membership on young preschoolers’ social preferences, estimates of similarity, and behavioral attribution. Collabra 2, 1–8. doi: 10.1525/collabra.44

[ref73] Schaafsma J. Williams K. D. (2012). Exclusion, intergroup hostility, and religious fundamentalism. J. Exp. Soc. Psychol. 48, 829–837. doi: 10.1016/j.jesp.2012.02.015

[ref74] Shweder R. A. (1997). The surprise of ethnography. Ethos 25, 152–163. doi: 10.1525/eth.1997.25.2.152

[ref75] Sierksma J. Thijs J. Verkuyten M. (2014). Children’s intergroup helping: the role of empathy and peer group norms. J. Exp. Child Psychol. 126, 369–383. doi: 10.1016/j.jecp.2014.06.002, PMID: 24999087

[ref76] Sierksma J. Thijs J. Verkuyten M. (2015). In-group bias in children's intention to help can be overpowered by inducing empathy. Br. J. Dev. Psychol. 33, 45–56. doi: 10.1111/bjdp.12065, PMID: 25252035

[ref77] Sparks E. Schinkel M. G. Moore C. (2017). Affiliation affects generosity in young children: the roles of minimal group membership and shared interests. J. Exp. Child Psychol. 159, 242–262. doi: 10.1016/j.jecp.2017.02.007, PMID: 28327384

[ref78] Tajfel H. Billig M. G. Bundy R. P. Flament C. (1971). Social categorization and intergroup behaviour. Eur. J. Soc. Psychol. 1, 149–178. doi: 10.1002/ejsp.2420010202

[ref79] Tajfel H. Turner J. C. (2004). “The social identity theory of intergroup behavior,” in Political psychology. eds. Jost J. T. Sidanius J. (New York, NY: Psychology Press), 276–293.

[ref80] Ting F. He Z. Baillargeon R. (2019). Toddlers and infants expect individuals to refrain from helping an ingroup victim’s aggressor. Natl. Acad. Sci. U.S.A 116, 6025–6034. doi: 10.1073/pnas.1817849116 PMC6442622 30858320

[ref81] Tooby J. Cosmides L. Price M. E. (2006). Cognitive adaptations for n-person exchange: the evolutionary roots of organizational behavior. Manag. Decis. Econ. 27, 103–129. doi: 10.1002/mde.1287, PMID: 23814325 PMC3693395

[ref82] Triandis H. C. (1990). “Theoretical concepts that are applicable to the analysis of ethnocentrism,” in Applied cross-cultural psychology. ed. Brislin R. (Newbury Park, CA: Sage Publications, Inc.), 34–55.

[ref83] Triandis H. C. (1994). Culture and social behavior. New York: Mcgraw-Hill Book Company.

[ref84] Turiel E. (1998). “Moral development,” in Handbook of child psychology. eds. Eisenberg N. Damon W., Social, emotional, and personality development. Vol. 3. (New York: Wiley), 863–932.

[ref85] Vezzali L. Stathi S. Giovannini D. (2012). Indirect contact through book reading: improving adolescents' attitudes and behavioral intentions toward immigrants. Psychol. Sch. 49, 148–162. doi: 10.1002/pits.20621

[ref86] Vezzali L. Stathi S. Giovannini D. Capozza D. Trifiletti E. (2015). The greatest magic of Harry potter: reducing prejudice. J. Appl. Soc. Psychol. 45, 105–121. doi: 10.1111/jasp.12279

[ref87] Vignoles V. L. Moncaster N. J. (2007). Identity motives and in-group favouritism: a new approach to individual differences in intergroup discrimination. Br. J. Soc. Psychol. 46, 91–113. doi: 10.1348/014466605X85951, PMID: 17355720

[ref88] Vorauer J. D. Sasaki S. J. (2009). Helpful only in the abstract? Ironic effects of empathy in intergroup interaction. Psychol. Sci. 20, 191–197. doi: 10.1111/j.1467-9280.2009.02265.x 19170943

[ref89] Walton G. M. Wilson T. D. (2018). Wise interventions: psychological remedies for social and personal problems. Psychol. Rev. 125, 617–655. doi: 10.1037/rev0000115, PMID: 30299141

[ref90] Walton G. M. Yeager D. S. (2020). Seed and soil: psychological affordances in contexts help to explain where wise interventions succeed or fail. Curr. Dir. Psychol. Sci. 29, 219–226. doi: 10.1177/0963721420904453, PMID: 32719574 PMC7384695

[ref91] Watson-Jones R. E. Legare C. H. Whitehouse H. Clegg J. M. (2014). Task-specific effects of ostracism on imitative fidelity in early childhood. Evol. Hum. Behav. 35, 204–210. doi: 10.1016/j.evolhumbehav.2014.01.004

[ref92] Watson-Jones R. E. Whitehouse H. Legare C. H. (2016). In-group ostracism increases high-fidelity imitation in early childhood. Psychol. Sci. 27, 34–42. doi: 10.1177/0956797615607205, PMID: 26573906

[ref93] Will G. Crone E. A. van den Bos W. Güroğlu B. (2013). Acting on observed social exclusion: developmental perspectives on punishment of excluders and compensation of victims. Dev. Psychol. 49, 2236–2244. doi: 10.1037/a0032299, PMID: 23544860

[ref94] Williams K.D. Case T.I. Govan C.L. (2003). “Impacts of ostracism on social judgments and decisions,” in Social judgments: Implicit and explicit processes, eds. Forgas J.P. Williams K.D. Hippel W. Von (New York: Cambridge University Press), 325–342.

[ref95] Williams K. D. Jarvis B. (2006). Cyberball: a program for use in research on interpersonal ostracism and acceptance. Behav. Res. Methods 38, 174–180. doi: 10.3758/BF03192765, PMID: 16817529

[ref97] Yamagishi T. Jin N. Miller A. S. (1998). In-group bias and culture of collectivism. Asian J. Soc. Psychol. 1, 315–328. doi: 10.1111/1467-839X.00020

[ref98] Yang L. Park Y. (2022). Group membership trumps shared preference in five-year-olds’ resource allocation, social preference, and social evaluation. Front. Psychol. 13:866966. doi: 10.3389/fpsyg.2022.866966, PMID: 35712199 PMC9197506

[ref1001] Yang X. Wu Z. Dunham Y. (2021). Children’s restorative justice in an intergroup context. Soc. Dev. 30, 663–683. doi: 10.1111/sode.12508

[ref99] Yazdi H. Heyman G. D. Barner D. (2020). Children are sensitive to reputation when giving to both ingroup and outgroup members. J. Exp. Child Psychol. 194:104814. doi: 10.1016/j.jecp.2020.104814, PMID: 32145479

[ref100] Yu J. Zhu L. Leslie A. M. (2016). Children's sharing behavior in mini-dictator games: the role of in-group favoritism and theory of mind. Child Dev. 87, 1747–1757. doi: 10.1111/cdev.12635, PMID: 28262934

[ref101] Zhao L. Heyman G. D. Chen L. Lee K. (2017). Praising young children for being smart promotes cheating. Psychol. Sci. 28, 1868–1870. doi: 10.1177/0956797617721529, PMID: 28898167

[ref102] Zinser O. Rich M. C. Bailey R. C. (1981). Sharing behavior and racial preference in children. Motiv. Emot. 5, 179–187. doi: 10.1007/BF00993896

